# Unveiling of Placental–Fetal Heart Interplay: A Novel Etiologic and Therapeutic Insight—A Narrative Review

**DOI:** 10.1155/jp/9100020

**Published:** 2026-03-23

**Authors:** Mohsen Shahidi, Arash Pooladi, Yousef Moradi

**Affiliations:** ^1^ Department of Pediatric Cardiology, School of Medicine, Kurdistan University of Medical Sciences, Sanandaj, Iran, muk.ac.ir; ^2^ Medical Genetics, School of Medicine, Research Institute for Health, Kurdistan University of Medical Sciences, Sanandaj, Iran, muk.ac.ir; ^3^ Health Metrics and Evaluation Research Center, Research Institute for Health Development, Kurdistan University of Medical Sciences, Sanandaj, Iran, muk.ac.ir

**Keywords:** fetal development, fetal heart, fetal placental circulation, placental disease

## Abstract

Congenital heart disease (CHD) is the most common fetal anomaly worldwide. The definite etiology of most CHD is not recognized. A direct genetic etiology is considered for a minority of patients. Most etiologies are attributed to epigenetic and environmental factors. Placental malformation is an overlooked cause of CHD that has recently received attention. This narrative review presents a hypothesis based on clinical reports and animal studies. The placenta and fetal heart have concomitant developmental regulatory pathways, and their diseases have a two‐way communication. Placental insufficiency may result in cardiac remodeling. Conversely, placental diseases are more frequent in association with fetal CHD. Fetal vascular malperfusion and genetic defects may play a role in placental and fetal heart disorders. Disturbed embryonic blood flow, such as syncytialization deformities and umbilical cord disorders, may lead to cardiac underdevelopment. Genetic, epigenetic, hormonal, and regulatory factors, including the NOTCH signaling pathway, SUMO‐modulated stress responses, and autophagy‐related genes, can affect both placental and fetal heart development. This novel information about the interplay between the placenta and fetal heart provides a new perspective on the etiologic factors of CHD and placental insufficiency. The current study aims to clarify the common causes of placental and fetal heart disorders.

## 1. Introduction

The placenta is the largest fetal organ and plays a crucial role in the growth of the fetal heart and organs [1]. During the first trimester, the placenta and fetal heart develop in tandem, known as the placenta–heart axis [2]. It is assumed that harmful alterations in the genetic expression of one organ can affect another during the first trimester [3]. After 12–14 weeks of gestational age, placental vascular resistance decreases as placental development progresses. Optimal placental and umbilical circulation influences normal fetal heart evolution [4]. The placenta develops by proliferation and fusion of villous cytotrophoblast (CTB) stem cells to produce the multinucleated syncytiotrophoblast (STB). The STB is responsible for transporting gas, nutrients, and the production of endocrine hormones to support fetal growth. It also plays a supporting role in *organogenesis and cardiovascular development*. Placental insufficiency is accompanied by inadequate trophoblast invasion. This leads to increased vascular resistance, insufficient nutrients, acceleration of hypoxia, alteration in the production of placental growth factors (PlGFs), hormones, or miRNAs, and fetal organ defects [1, 4]. CHD has a high rate of association with placental abnormality. CHD is present in more than 70% of chronic placental histopathologic lesions. The severity of CHD has a positive correlation with placental deformities. *Furthermore, shared genetic and hormonal defects between the placenta and fetal heart may lead to CHD and placental disorders*. Additionally, gene defects in one organ may indirectly affect another [3].

## 2. Methods

This narrative review was conducted to synthesize recent evidence on the interplay between placental development and fetal heart defects, with a focus on implications for therapeutic strategies. To provide a timely analysis that balances comprehensiveness with efficacy, the rapid review approach was chosen.

### 2.1. Search Strategy

A focused search was performed using the PubMed and Scopus databases, covering studies published between 2017 and 2024. The search utilized specific keywords, including “placentation,” “placenta development,” “placental dysfunction,” “syncytialization,” “fetal heart defect,” “fetal heart dysfunction,” and “fetal congenital heart disease.” This strategy is aimed at capturing the most relevant and up‐to‐date research.

### 2.2. Screening and Selection Process

Initial screening was conducted based on abstracts to manage the large volume of retained articles.

Articles were selected according to the following criteria:

*Inclusion criteria*: These consist of original research articles published in peer‐reviewed journals, full‐text articles available, and studies directly addressing the relationship between placental issues and fetal heart defects.

*Exclusion criteria*: These include studies that did not link placental and fetal heart conditions, editorials, case reports, and articles without full‐text access.

### 2.3. Data Extraction

Forty‐one articles were considered eligible for the review after applying the inclusion and exclusion criteria. Data from these articles were extracted systematically, focusing on the nature of placental dysfunctions, fetal heart defects, and any associated therapeutic implications. The extracted data were then analyzed to identify key themes and trends relevant to the objectives of the reviews.

### 2.4. Synthesis Data

The data was synthesized with an emphasis on rapidly generating insights. The findings were organized to highlight the connection between placental dysfunction and fetal heart defects, with a focus on drawing conclusions that could inform therapeutic policy.

## 3. Results

### 3.1. Companionship of Fetal Heart and Placenta

#### 3.1.1. Parallel Development of Placenta and Fetal Heart

There is a concomitant organogenesis of the fetal heart and placenta. The heart tube differentiates into the embryonic heart at the third week of gestational age. Vitelline and chorionic circulations are the fetal–placental blood vasculatures [5]. Shared genetic factors control the coevolution of the fetal heart and placenta. Harmful alterations of genetic expression in one organ can affect another during the first trimester. The fetal heart is the forerunner organ originating from the mesoderm that undergoes a subsequent developmental process. The asymmetric nature of cardiac development contributes to the complexity of molecular events during the first trimester. Primary and secondary placental villi originate from trophectoderm and extraembryonic mesoderm. Simultaneously, the primitive cardiac structure is progressing. First, angiogenic precursor cells originate from mesenchymal cells inside the villi and finally differentiate into the placental vasculature. The primitive heart is irrigated primarily by the secondary yolk sac and later by the aboriginal placental circulation [3]. The vitelline circulation is the primary fetal vascular flow and generates primitive fetal heart development. The fetal chorionic arteries diverge into the umbilical arteries to feed the placental chorionic villi. At the end of the first trimester, lower vascular resistance creates remodeling and full development of the placental vasculature [5].

#### 3.1.2. Similarities of the Cardiac Endocardial Cells (ECs) and the Placental Trophoblastic Structures

Endothelial cells (ETs) in the heart have multipotential capability to create various cardiac structures, including valves, coronary endothelium, liver vasculature, fat cells, mural cells, and blood cells [6]. On the other hand, there are similarities between cardiac ETs and placental ETs and their trophoblastic structures, especially extravillous trophoblasts (EVTs). There are already 128 shared expressed genes between cardiac ETs and placental CTB. Likewise, the fetal heart ETs already have 197 shared expressed genes with placental EVTs, 128 with CTBs, and 80 with STBs during the first trimester. Genes such as FLT1, GATA2, ENG, and CDH5 are involved in this process [**3** **]**. Further, there are commonly expressed genes between cardiomyocytes and placental trophoblastic structures (EVTs, CTBs, and STBs). During the first trimester, there are only 16 shared expressed genes between fetal cardiomyocytes and placenta ETs, including glucose transporter SLC2A1, AK1, CAV1, and BEX1 [3].

### 3.2. Shared Genetic and Hormonal Defects Between the Placenta and Heart May Contribute to Their Common Disorders

**Figure 1:**
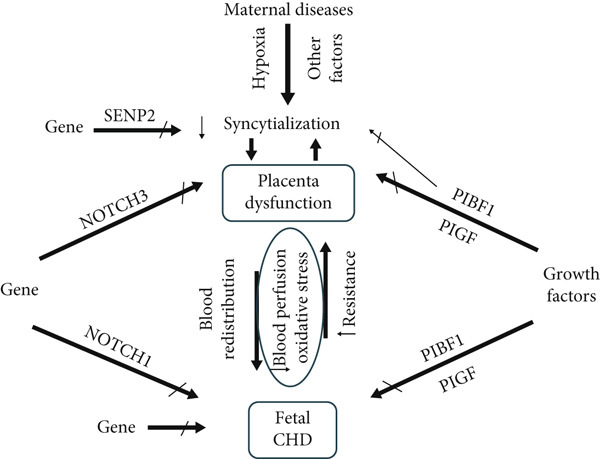
Diagram of placenta and fetal heart interaction (P‐FH): Blood perfusion disorder and oxidative stress have bidirectional interaction between the placenta and fetal cardiovascular system. Genes and growth factors have positive developmental effects on both the fetal cardiovascular system and the placenta. Interplaying hemodynamic factors of the fetoplacental axis are indicated by to and fro large arrows between placenta dysfunction and fetal CHD. NOTCH 1 and 3 are receptors of the NOTCH gene on the endocardium and cytotrophoblast, respectively. PIBF1: progesterone immunomodulatory binding factor 1, PlGF: placental growth factor, SUMO: small ubiquitin‐related modifier, SENP2: (SUMO)‐specific protease 2.

Recent studies reported more than 328 common gene expressions between cardiac and placental ETs. Placental and fetal heart disorders may be due to a single origin of genetic, epigenetic, or hormonal defects [6–8]. Wilson et al. reported shared genes and cellular pathways between the placenta cells and fetal heart, suggesting that their defects may contribute to the development of CHD and placental dysfunction (Figure 1) [3].

#### 3.2.1. NOTCH Signaling Pathway

The NOTCH family of genes plays a pivotal role in both placental and cardiovascular development. Notch receptor 1 (NOTCH1), expressed in the fetal endocardium, regulates cardiogenesis, and NOTCH3, found in CTB progenitors, supports placental vascular maturation and syncytialization. Defective NOTCH signaling can disrupt placental implantation and fetal cardiac structure [6, 9].The NOTCH groups control EVT differentiation, which is the starting part of the trophoblast invasion pathway. NOTCH1 is specifically expressed in the villi and is the core regulator of EVT development. NOTCH2 regulates EVT migration, differentiation, and spiral artery remodeling. NOTCH3 is particularly present in CTB progenitors, and its active form, the nuclear NOTCH3 intracellular domain (NOTCH3‐ICD), interacts with the transcriptional coactivator mastermind‐like 1 (MAML1) to inhibit differentiation into multinuclear STBs. The NOTCH3‐ICD regulates trophoblast stem cell (TSC) differentiation. Villous CTB progenitors exclusively express the NOTCH3 receptor, whereas TSCs exhibit an additional low level of NOTCH1. Furthermore, NOTCH1 expression is present in the EVT progenitor pool of anchoring villi. NOTCH1‐ICD plays a role in the formation of EVT progenitors [9]. NOTCH pathways are also involved in the development of CHD. Eight of the 26 genes involved in heart and placental vasculature defects have similar phenotypic presentation. This includes NOTCH1 and its receptor ligand DLL4. Notch signaling regulates the morphogenesis of the cardiac cells, chambers, and valves. Additionally, CHD may be linked to mutations in notch pathway genes, including NOTCH1, NOTCH2, DLL4, JAG1, and MAML215. Mutations in NOTCH1 create a variety of CHD, ranging from the bicuspid aortic valve to HLHS (Table 1) [3].

**Table 1:** Key genes and their functions in placental and fetal heart development.

**Gene**	**Function**	**Clinical impact**	**Reference**
NOTCH1	Regulates endocardial development and cardiogenesis	Defects lead to congenital heart defects (CHDs) and impaired fetal heart chamber formation	[1, 2]
NOTCH3	Controls and promotes cytotrophoblast differentiation, proliferation, and placental and vascular maturation	Abnormalities contribute to placental insufficiency (abnormal placental implantation and vascularization)	[1, 2]
SENP2	Modulates SUMOylation, regulating trophoblast proliferation, differentiation, and syncytialization	Deficiency results in fetal heart malformations and placental insufficiency	[3]
SUMO2/3	Maintains protein homeostasis and stress response	Dysregulation exacerbates oxidative stress, impairing placental and heart function (leading to CHDs)	[3, 4]
LIN28A/B	Regulates placental syncytialization and embryonic organogenesis	Downregulation linked to preeclampsia and CHDs	[5]
PlGF	Supports angiogenesis and fetal heart growth	Deficiency impairs cardiac remodeling, vasculogenesis, and placental function	[6–9]
PIBF1	Enhances trophoblast syncytialization and cardiovascular development	Low levels associated with fetal heart defects and pregnancy complications	[10]
TBX3	Influences trophoblast lineage decisions	Essential for normal placental structure and cardiac morphogenesis	[11]
KLF15	Enhances placental cell proliferation and inhibits oxidative stress	Deficiency linked to preeclampsia, fetal cardiac stress, and placental insufficiency	[12]
Autophagy‐related genes	Control degradation of damaged proteins and organelles	Defective autophagy leads to oxidative stress and tissue dysfunction	[13]

#### 3.2.2. SENP2 and SUMO Pathways

Small ubiquitin‐related modifier (SUMO)–specific protease 2 (*SENP2*) regulates trophoblast proliferation and differentiation, which is encoded by the gene. SENP2 has a principal role in the normal development of the placenta and TSCs [10]. SENP2 deficiency leads to abnormal trophoblast layers and impaired fetal heart anatomy. SUMO proteins modulate cellular stress responses, and their dysregulation affects placental and cardiac development [10]. The SENP2 role in placentation is related to protein regulation by SUMO2 and SUMO3. SENP2 controls SUMO1 adjustment of Mdm2 and p53 activities for the vital placental trophoblast layers′ development. This process creates regular placentation and fetal organogenesis. “SUMOylation” is the process of the transformation of SUMO polypeptides to their targets. SUMO‐specific proteases are involved in the reversed “deSUMOylation” process that removes SUMO. SENP2 deficiency leads to severe placental underdevelopment associated with heart and brain defects and embryonic death. Inactivation of the gene encoding SUMO2 or SUMO3 (reducing the level of SUMO modifiers) relieves the placental insufficiencies caused by the loss of SENP2 to recover the abnormal trophoblast layer development (due to hyper‐SUMOylation and loss of SENP2). This will improve the proper formation of the heart structures, including the atrioventricular cushion and myocardium (Table 1) [10]. Some reports suggested that those placental gene expression patterns related to STB‐1 are more associated with fetal heart diseases [1, 11, 12].

#### 3.2.3. LIN28A and LIN28B

These proteins bind to mRNA to coordinate embryonic development and may have a regulatory role in the development of trophoblast and its syncytialization. Consequently, their insufficiency may contribute to preeclampsia (PE) [13]. They coordinate embryonic and placental development. Their downregulation has been linked to PE and increased CHD risk due to impaired syncytialization and trophoblast migration (Table 1) [13].

#### 3.2.4. Endothelial Nitric Oxide Synthase (NOS3)

NOS3 regulates placental and cardiac angiogenesis and vascular development. NOS3 gene defects are associated with an increased risk of complications in pregnancy, such as PE and cardiac underdevelopment. Polymorphisms in NOS3 have been reported in association with sporadic CHD, specifically perimembranous ventricular septal defects [3].

#### 3.2.5. Epigenetic Modifications

Epigenetic alterations, such as changes in histone acetylation and DNA methylation, impact gene expression critical for placental and fetal heart development. For instance, epigenetic silencing of angiogenic genes may contribute to PE and subsequent cardiac remodeling in the fetus [14].

#### 3.2.6. Genes Regulating Autophagy and Stress Response

Autophagy (degradation of aggregated proteins by autophagosomes) is a protective gene‐dependent mechanism. The autophagy mechanism is responsible for clearing the damaged organelles and preventing oxidative stress and inflammation, which are all involved in placental and fetal heart disorders (Table 1) [15]. Defects in autophagy‐related genes can lead to protein aggregation in placental and cardiac tissues, resulting in multiorgan disease. Defects in autophagy may cause heart failure, PE, and fetal growth restriction (FGR) [15].

#### 3.2.7. Peroxisome Proliferator‐Activated Receptor Gamma (PPAR*γ*)

PPAR*γ* is a nuclear receptor that plays a role as a transcriptional coregulator of genes involved in mitochondrial activities. Ling Lai et al. emphasized the angiogenic effects of PPAR*γ* by a tube formation assay of cultured human umbilical vein endothelial cells (HUVECs). PPAR*γ* induces angiogenesis and supports endothelial function through the secretion of angiogenic proteins. Reduction in PPAR*γ* contributes to the placental dysfunction, which normally activates placental function and trophoblast differentiation [16].

The role of PPAR*γ* in the development of the placental–fetal heart axis has been confirmed in PPAR*γ*‐null mice. The research history of PPAR*γ* in the placenta is older, though it also plays a role in fetal heart development. In PPAR*γ*‐null mice models, placental labyrinth development was defective, and there was a thinning of ventricular myocardium. However, mortality was lower in those treated through tetraploid aggregation. Thus, PPAR*γ* is one of the shared factors in the placental–fetal heart axis [11].

#### 3.2.8. Hydrogen Sulfide

Enzymatic downregulation of hydrogen sulfide resulted in the remodeling of the placental vasculature. Treatment with a hydrogen sulfide donor prevented these deformities [5, 17].

#### 3.2.9. Hormonal Factors

Current studies suggest that growth factors such as angiogenic molecules and transcription factors have equal effects on both placental and cardiovascular development [18]. *PlGF*, a member of the vascular endothelial growth factor (VEGF) family, is suggested as a regulatory factor of cardiac remodeling by activating cardiac fibroblasts (Figure 1). The suppressor of cytokine signaling 3 (SOCS3) and the protein tyrosine phosphatase nonreceptor type 2 (PTPN2) regulate the decidualization of the endometrium (differentiation of stromal cells into decidual cells), which provides embryonic implantation. Progesterone immunomodulatory binding factor 1 (*PIBF1*) can activate this process (Table 1) [19]. Likewise, they are involved in the formation of the placental vascular network. Placental complications like PE and FGR dysregulate the VEGF and PIG that are commonly associated with CHD [7, 8]. PIGF is an angiogenic agent with both cardiogenic and vasculogenic roles during fetal heart development. Cardiogenic growth factors play a substantial role in heart development. Nevertheless, its definite role in cardiogenesis is not yet clear [20]. Jones et al. conducted an animal study to treat placental insufficiency and FGR. They reported successful results via a polymer‐based nanoparticle with transient gene expression of human insulin‐like 1 growth factor (hIGF1) in placental trophoblast (Table 1) [21]. Lee et al. found that endogenous *PIBF1* probably controls the syncytialization. It has a fundamental role in cell proliferation. Further, PIBF1 promotes placental vasculature (the connection between STB and adjacent vascular cells) and fetal heart development. Low levels of PIBF1 may be associated with abortion, eclampsia, and fetal heart defects (Figure 1) [4].

### 3.3. Placental–Fetal Hemodynamic Disturbances May Contribute to the Development of Fetal CHD and Placental Dysfunction

#### 3.3.1. Etiologic Factors of Hemodynamic Disorders

Hemodynamic problems can also influence fetal organ malformation, including CHD. Fetal vascular malperfusion (FVM) is due to blood flow obstruction to or from the placenta. It may be partial or complete and acute or intermittent [12]. The function of the normal placenta is to supply fetal oxygen and nutrients, hormonal and immunologic regulation, and filtration of toxins. [7] CHD is associated with different placental defects, including decreased weight, shallow depth of invasion, irregular location, and shape [12]. Further, chorangiosis, maternal vascular malperfusion and FVM, fetal thrombotic vasculopathy, chronic inflammation, delayed villous maturation with edematous villi, and increased nucleated red blood cells are other placental deformities associated with CHD [7, 22]. Umbilical cord hypercoiling, true knots, and shortened or elongated umbilical cords could create umbilical cord obstruction and FVM. The umbilical vein compresses more easily. Additionally, CHD has an association with abnormal umbilical cord insertion and a single umbilical artery, possibly due to the reduction of oxygen and nutrient transportation [22]. FVM can result in stillbirth, cerebral palsy, and CHD. Hypertension with or without PE, maternal diabetes mellitus, and hypercoagulable states are some predisposing factors of FVM. Thrombosis, avascular chorionic villi, intramural fibrin, and stromal–vascular karyorrhexis are pathological findings of FVM placentas [12]. In animal models, induced hemodynamic changes in the umbilical vein and artery led to placental remodeling and CHD [3].

#### 3.3.2. Pathophysiology of FVM

Placental malperfusion parallels the reduction of wall shear stress (WSS). Morley et al. indicated that blood flow shear stress (FSS) preserves placental hemostasis and perfusion by releasing endothelial nitric oxide and generating vasodilation [23]. Barnett et al. demonstrated that Piezo1 ion channels in fetoplacental endothelium are FSS‐mechanosensitive proteins. A new therapeutic approach is to trigger these receptors with agonist agents (Yoda1) [24, 25]. Semmler et al. recognized that there is a concurrence between fetal cardiac changes and the development of PE. Right ventricular systolic dysfunction is probably the result of increased placental resistance and cardiac afterload. Additionally, left ventricular diastolic dysfunction is possibly owing to the redistribution of fetal circulation after hypoxia (Figure 1) [26]. FVM may induce hypoxia. Hypoxia can lead to end‐organ underdevelopment by activating inflammatory phenomena and disrupting angiogenesis [7]. In FGR, placental insufficiency and subsequent reduction of oxygen and nutrient supply can disrupt cardiomyocyte growth and create cardiovascular defects. Further, villous hypoplasia and thrombosis increase placental resistance and cardiac afterload. Genetic, epigenetic, and hemodynamic abnormalities could contribute to placental deformity and CHD [7].

#### 3.3.3. CHD Causes Placental Underdevelopment

CHD may be associated with hemodynamic changes and hypoxia in the fetal circulation [7]. Leon et al. reported that fetal CHD induces placental malperfusion and microscopic changes. They believe that, in CHD, both malperfusion and abnormal oxygen delivery can result in placental and brain underdevelopment [27]. Sufficient placental perfusion is vital for optimal syncytialization (Figure 1). WSS can activate the upregulating STB‐specific genes and downregulating CTB‐specific genes [28]. Studies have shown that the severity of heart failure is related to edematous villi and increased nucleated red blood cells in villous vessels [22].

## 4. Discussion

A successful pregnancy depends on the proper development of the placenta, which is the normal proliferation, differentiation, and implantation of trophoblasts. Genetic and hormonal pathways control this process. The STBs are a maternal–fetal circulatory barrier responsible for oxygenation, hormonal secretion, and the transportation of nutrients and waste products. Abnormal placentation and defective spiral artery maturation can lead to malperfusion with subsequent oxidative stress and obstetrical syndromes, including spontaneous abortion, preterm labor, FGR, and PE [9]. The abnormal structure and hemodynamics of the placenta influence regular fetal heart development [5]. STB layer defects might be the principal placental etiology that induces fetal heart disorders. Uteroplacental malperfusion (e.g., in PE or FGR) leads to proliferative and deformed placental arteries and a subsequent increase in umbilical resistance. Increased fetal heart afterload likely leads to cardiac underdevelopment (Figure 1) [29].

Successful placental implantation depends on normal syncytialization by trophoblast cell remodeling and invasion into the endometrium. The placental pathologic and biologic changes include abnormal villous maturation and abnormal levels of angiogenic or antiangiogenic agents in maternal and umbilical blood [30]. Chorionic vessel changes, including thrombosis, chorangiosis, infarction, and hypomature villi, increase the risk of CHD by up to six times. These abnormalities affect antegrade umbilical vein or retrograde umbilical artery flows. In animal studies, disturbed embryonic blood flow correlated with cardiac underdevelopment (e.g., hypoplastic left heart syndrome), lower placental weight, and fetal growth retardation [5, 7].

### 4.1. Maternal Comorbidities May Cause Fetal Heart Disease

Pregnancies complicated by hypertension, diabetes mellitus, PE, chronic deciduitis, substance abuse, and alcohol consumption have a higher rate of CHD. Prepregnancy hemoglobin A1c serum level is significantly correlated with the frequency of CHD. Pregestational and gestational diabetes mellitus are both associated with an overabundance of placental villous structures and their capillaries identified as chorangiosis [12]. Environmental chemicals, medications, maternal malnutrition, pregnancy comorbidities, and genetic defects may interrupt the normal placental–fetal heart developmental process [22]. Placenta‐induced fetal CHD may occur through the following mechanisms: *hemodynamic disorders, hypoxia, malnutrition, chronic deciduitis, endocrine abnormalities, and placenta-produced growth factors.* Many of these complications are due to abnormal placentation or disturbed genetic pathways [22]. Recent studies indicated that fetal CHD conversely increases the risk of placental underdevelopment, mostly PE. These indicate the interrelationship of fetal heart and placental development and the association of CHD with PE, known as the “great obstetrical syndromes.” [2] During the first trimester, harmful alterations of genetic expression in one organ can affect another [3].

#### 4.1.1. PE

PE is known as hypertension and proteinuria (or end‐organ dysfunction) that begins after 20 weeks of gestation. It is a multisystemic disease with acute and long‐term fetomaternal complications, including fetal cardiovascular disorders. The proposed pathophysiology is the release of the angiogenic factors due to placental insufficiency [5]. Abnormal generation of angiogenic proteins by aberrant trophoblasts leads to systemic endothelial damage. Inadequate invasion of the trophoblast into the endometrium, inefficient EVTs migration, deficient spiral artery remodeling, and increased uteroplacental vascular resistance are probably the initial steps of the disease leading to progressive uteroplacental hypoxia. Reduced placental blood flow contributes to oxidative stress and the release of toxic placental factors, which lead to placental damage, mitochondrial dysfunction, and immunological processes. The ongoing process is the release of vasoactive components, including oxidative stressors, proinflammatory factors, and cytokines, into the maternal circulation. These reactive factors cause systemic ET damage and generalized vascular hypercoagulability [31]. Developing PE induces mild fetal left ventricular myocardial dysfunction with minimal clinical manifestation [32]. Auger et al. and a Norwegian study reported more severe CHD, including atrioventricular septal defects in early‐onset PE (before the 34th week). All of the above support the important role of cardio–placental axis interaction [5]. Jones et al. reported that hypoplastic left heart syndrome is associated with abnormal placental morphology [33]. Early and late embryonic hypoxia and oxidative stress lead to cardiac remodeling or cardiovascular disease through decreased activity and expression of mitochondrial proteins (Figure 1) [34]. Ferreira et al. reported that impaired angiogenesis is associated with PE and CHD. They declared that different vasculogenic pathways are related to CHD [35]. Animal models proposed that hypoxia is the core etiology of associated CHD by influencing the proliferation of multipotent second heart field progenitor cells. Current studies have not yet suggested a mechanism‐targeted medication for CHD associated with PE. Nonetheless, there are many reported therapeutic strategies for PE, which may prevent CHD [12].

#### 4.1.2. FGR

CHD is often associated with fetal growth abnormalities. The definition of FGR is an estimated fetal weight of < 10th percentile and occurs in up to 10% of pregnancies [21]. FGR is usually due to placental insufficiency, suboptimal uteroplacental perfusion, and hypoxia with early and late‐onset cardiovascular complications [36]. Placental insufficiency is due to inadequate trophoblast invasion and subsequent high placental vascular resistance, resulting in decreased oxygen and nutrient supply and an increased risk of CHD and FGR [21]. Cardiovascular remodeling is associated with cardiac and placental mitochondrial dysfunction and may persist until adulthood. An animal model investigation showed that early‐onset hypoxia increases only uterine artery resistance (but not that of the umbilical artery). Early‐ and late‐onset hypoxia influence placental insufficiency, FGR, and fetal cardiac diastolic dysfunction [37]. Zhang et al. declared that FGR is associated with evidence of early cell apoptosis, autophagy, and compensatory hypertrophic responses of cardiomyocytes in the fetal heart. This process is due to placental restriction and hypoxia [38]. This may have similarities with maternal diseases like diabetes and PE, which induce placental dysfunction and increase the risk of fetal CHD (Figure 1) [5].

#### 4.1.3. Placental Inflammatory Disease and CHD

Chronic deciduitis occurs in the presence of maternal immune activation, specifically idiopathic villitis and chronic chorioamnionitis [12]. Nonetheless, chronic deciduitis may be secondary to chronic endometritis that is linked with recurrent miscarriage. The association of the high rate of heart defects with miscarriage raises the suspicion that chronic deciduitis impacts fetal heart development [12]. In animal models, induced neutrophil‐driven placental inflammation led to placental underdevelopment associated with migration of maternal inflammatory monocytes to the embryonic heart and disrupted cardiogenesis [12]. Some inflammatory placental diseases may lead to under‐ or overinvasions of EVTs. Both under‐ and overinvasions are associated with abnormal placental perfusion that may be immune‐induced. Disturbed maternal–placental blood flow causes prominent embryonic cardiac development, indicating a robust combination of placental and cardiac deformities. Placenta accreta is an obvious example of EVT overinvasion, which may lead to placental malperfusion and cardiac underdevelopment [3].

#### 4.1.4. In Vitro Fertilization (IVF)

Huluta et al. reported that, in IVF, there is evidence of fetal cardiac remodeling (globular left ventricle with decreased systolic function) at midgestation, which is unrelated to the utilization of fresh or frozen embryos. The progression or continuation of these primary changes is still unclear [39].

### 4.2. Paraclinical Evaluation of Fetoplacental Perfusion

*Cardiovascular profile (CVP) is the scoring system used to measure the associations between placental macro- and microscopic abnormalities and the severity of fetal heart failure* [22]. New MRI techniques may indicate the relationship between placental development, placental insufficiency, and fetal cardiovascular physiology. It reveals both structural and functional characteristics of the fetal cardiovascular system [5, 12]. Fetal Doppler evaluates placental function through secondary measurements of the umbilical artery and fetal cerebroplacental ratio, with limited effectiveness in distinguishing between CHD and normal fetal circulation [12].

Serial evaluation of the placenta and fetal heart could be reasonable in the following conditions:

a. Pregnancy comorbidities: The presence of maternal comorbidities like diabetes, hypertension, PE, endometritis, deciduitis, fetal thrombotic vasculopathy and infarction, chorangiosis, chronic inflammation, and decreased placental weight may induce placental and fetal heart diseases.
b. Reduced maternal–fetal blood supply:
  1. Syncytialization disorders indicated by FGR, PE, previous fetal demise, and history of high‐risk pregnancies may threaten maternal–fetal blood exchange
  2. Umbilical cord abnormalities
  3. Fetal cardiovascular failure and hydrops fetalis

### 4.3. Supposed Therapies

#### 4.3.1. Nonviral, Polymer‐Based Nanoparticle

A new introduction of a nonviral, polymer‐based nanoparticle facilitates the delivery and transient gene expression of hIGF1 in placental STB. It is used as a therapeutic method for placental insufficiency and FGR to restore appropriate placental function [21].

#### 4.3.2. Epigenetic Therapies

Histone deacetylase inhibitors may reverse pathological gene expression and improve placental–fetal cardiovascular health [14].

#### 4.3.3. NOTCH Pathway Activation

Chi et al. reported that the histone methyltransferase G9a, also known as Ehmt2, endorses placental vascular maturation by activating the NOTCH pathway. NOTCH pathway activation in G9a deficiency improves placentation [40].

#### 4.3.4. Transamniotic Stem Cell Therapy (TRASCET)

In animal models, TRASCET reduces fetal heart dysfunction and inflammation and improves FGR through mesenchymal stem cells (MSCs). Human trophoblast stem cells (hTSCs) influence placental and fetal nutrition during syncytialization [41].

#### 4.3.5. PPAR*γ*

PPAR*γ* is decreased in PE. It is necessary for trophoblastic differentiation. Grimaldi et al. reported enhanced angiogenesis potential of ETs through activation of PPAR*γ* to improve placental and endothelial function in PE [16].

## 5. Conclusion

There is a close association between placental abnormalities and fetal cardiovascular underdevelopment. Placental insufficiency is at least responsible for some kinds of CHD. Conversely, CHD may contribute to placental deformity [2]. Additionally, the fetal heart and placenta share common genetic factors and signaling pathways that have synergistic and parallel effects for their development. Upcoming human and animal model investigations must reveal blurred aspects of placenta–heart relationships and their relevant therapies.

## Conflicts of Interest

The authors declare no conflicts of interest.

## Funding

No funding was received for this manuscript.

## Data Availability

The findings of this study are available from the corresponding author and can be accessed upon reasonable request.
